# Elimination and detection of viruses in meristem-derived plantlets of sweetpotato as a low-cost option toward commercialization

**DOI:** 10.1007/s13205-012-0080-6

**Published:** 2012-08-08

**Authors:** Iftekhar Alam, Shamima Akhtar Sharmin, Mst. Kamrun Naher, Md. Jahangir Alam, Mohammad Anisuzzaman, Mohammad Firoz Alam

**Affiliations:** 1 Division of Applied Life Sciences (BK21), Gyeongsang National University, Jinju, 660-701 Republic of Korea; 2 Department of Botany, University of Rajshahi, Rajshahi, 6205 Bangladesh; 3 Department of Bioscience (Integrated Bioscience Section), Graduate School of Science and Technology, Shizuoka University, Shizuoka, 422-8529 Japan

**Keywords:** Meristem, Sweetpotato, Grafting, *Ipomoea setosa*, Viral disease

## Abstract

Viral diseases affecting sweetpotato are the most devastating and cause up to 98 % yield loss. In this paper, we report, meristem culture, graft transmission and virus indexing for management of viral pathogens in seven elite sweetpotato cultivars. Plantlets were developed in vitro from the apical meristematic dome with one to two leaf primordia. Mericlones were grafted on virus-sensitive indicator plant *Ipomoea setosa* and no viral disease symptoms were seen on *I. setosa* leaves in most cases. This indicates that no viruses translocated from meristem-derived scions to the virus-sensitive root stock. On the other hand, most of the non-tested traditional planting material induced distinct disease symptoms upon grafting, which revealed the presence of one or more viruses in it. About 85 % of mericlones recovered from 0.3–0.5 mm size meristem were tested as virus free, whereas it is difficult to culture meristems smaller than 0.3 mm due to dissection damage and too small a size. Virus-tested mericlones were further micropropagated and transferred to the field. Only few plants were found to be diseased in the R1 field trial. Root yield in the R2 generation was increased significantly when compared with non-tested control plants. During field exposure, only a low percentage of healthy plants were found infected with viruses when managed in a net house. This implies that viral vectors were present during the growing season and reinfection could be effectively reduced by net house management. We concluded that this low-cost technique of producing virus-tested planting material would significantly boost the yield through efficient removal of yield-reducing pathogens.

## Introduction

Sweetpotato (*Ipomoea batatas*) is one of the world’s most important, versatile and underexploited food crop that ranks seventh in the world in terms of total production (FAOSTAT 2008). High yields, lower agricultural input and rich nutrients, primarily of carbohydrates, make it one of the staple foods for millions of people, especially in developing countries. The remarkable provitamin A qualities of orange-fleshed types make it an immediate solution to combat vitamin A deficiency in Sub-Saharan Africa (Woolfe 1992). Viral disease is considered as one of the most important cause of yield loss and cultivar decline. Among the 11 well-recognized sweetpotato viruses, sweetpotato feathery mottle virus (SPFMV; *Potyvirus*) has a pervasive distribution, while the others are localized to one or more geographic areas (Moyer and Salazar 1989; Kreuze et al. 2000; Mukasa 2004). Viruses strains of SPFMV coupled with its ubiquitous nature hindered the identification of many other viruses. In Bangladesh, at least five sweetpotato viruses have been reported (BARI 2003). Multiple infection and synergism are common in sweetpotato. Sweetpotato viral disease (SPVD), caused by the synergistic interaction of sweetpotato feathery mottle virus and sweetpotato chlorotic stunt virus, reduces yield by up to 98 % (Mwanga et al. 2002; Clark et al. 2012). Besides this, chlorotic dwarf, *Camote Kulot* and some other complex infections exist (Di Feo et al. 2000; Salazar and Fuentes 2000). Virtually all sweetpotatoes grown from non-virus-tested materials revealed the presence of one or more viruses in them (Moyer and Salazar 1989).

Accumulation of viruses and diseases occurs in sweetpotato through the adventitious, root-to-sprout propagation method used in commercial production. In most regions including Asia and USA, the subsequent season’s sweetpotato crop is produced by using adventitious sprouts of ‘seed’ roots saved from the previous crop. Saved ‘seed’ roots of sweetpotato plants that became infected with viruses produce virus-infected adventitious sprouts during the growing season. Continuous use of virus-infected planting material may lead to cultivar decline. Moreover, some of the viruses have insect vectors which increase the rate of reinfection in the growing season on availability of local inoculum and favorable environment. Over time, the entire population of a given clonal variety could be infected with the disease. Even without visible symptoms, infected plants exhibit reduced growth and yield performance, and could spread the disease to non-target varieties. SPVD epidemics have been, in many cases, associated with the disappearance of a former elite cultivar (Gibson et al. 1997). Yield loss due to viral diseases was estimated to be 15–48 % in China, 34–97 % in Egypt (Salazar and Fuentes 2000), 50 % or more in Israel (Milgram et al. 1996) and 80–98 % in East Africa (Mwanga et al. 2002; Wambugu 2003). Quality was also affected by alterations in the shape and skin color of storage roots. The lack of resistant genotypes makes clean planting material the only immediate straightforward solution to increase the yield and to maintain the production areas. Controlling sweetpotato viral diseases is one of the top research priorities of CIP by adopting virus-free seed program to reduce this production constraint in developing countries (Zhang and Salazar 2000). Current research has demonstrated significant benefits in yield and quality using pathogen-tested planting material when compared with farmers’ traditional non-tested material (Carey et al. 1999; Fuglie et al. 1999; Zhang and Salazar 2000; Carroll et al. 2004). Moreover, high possibility of healthy materials necessitates the importance of continuous use of certified, virus-tested seed roots or cuttings (Ling et al. 2010).

Plant meristem culture is a unique technique to free away various pathogens including viruses, viroides, mycoplasma, bacteria and fungi (Walkey 1978; Pierik 1989; Bhojwani and Razdan 1996). Meristems are frequently devoid of systemic pathogen due to the absence of differentiated conducting tissues. In addition, the use of planting material derived from pre-existing meristems has been proposed to reduce the amount of variation among the propagules and to retain genetic integrity (Villordon and LaBonte 1996). Therefore, its application may help to slow down the process of cultivar decline due to accumulation of viruses and mutations. Reports have been published on successful meristem culture and virus indexing in sweetpotato and other crops over two decades (Frison and Ng 1981; Dagnino et al. 1991; Alam et al. 2004). Nevertheless, sweetpotato improvement through virus-indexed mericlones is important for unlocking yield potential of diversified elite genotypes grown under various agro-ecological zones and cultural practices by using disease-free and uniform propagules. However, sweetpotato has a very wide genetic base and highly heterogeneous tissue culture response. Therefore, improvement of diverse elite genotypes grown under various agro-ecological zones and cultural practices through virus-indexed and uniform mericlones is important for unlocking yield potential.

Virus detection is a routine work for virus-free planting material production and safe movement of germplasm. Serology or other molecular diagnoses are expensive for many developing countries. *Ipomoea setosa* is a nearly universal sensitive indicator plant for sweetpotato viruses, which is used for graft-transmitted virus detection. Current international guidelines document that graft indexing successfully reveals most sweetpotato viruses (Moyer et al. 1989; Laurie et al. 2000; Loebenstein et al. 2003; Mukasa et al. 2003). Moreover, SPFMV is often present at a concentration below the limit of detection by ELISA (Winter et al. 1992; Vetten et al. 1996; Aritua et al. 1998; Gibson et al. 1998; Karyeija et al. 2000) and, in those cases, can be detected only by grafting onto *I. setosa* instead of serological assay (Gutiérrez et al. 2003). Therefore, research institutes and seed enterprises of developing countries could benefit from using this technique for routine monitoring of planting materials in an inexpensive way without employing highly skilled manpower. In this report, we developed a protocol for meristem culture and micropropagation for several elite sweetpotato cultivars. We also show the effectiveness of graft-transmitted virus-indexing system of mericlones and their field management as a means of suitable and low-cost protocol for producing virus-free sweetpotato planting material for subtropical and warm temperate environmental conditions where viral diseases are very frequent.

## Materials and methods

### Meristem culture

**Fig. 1:**
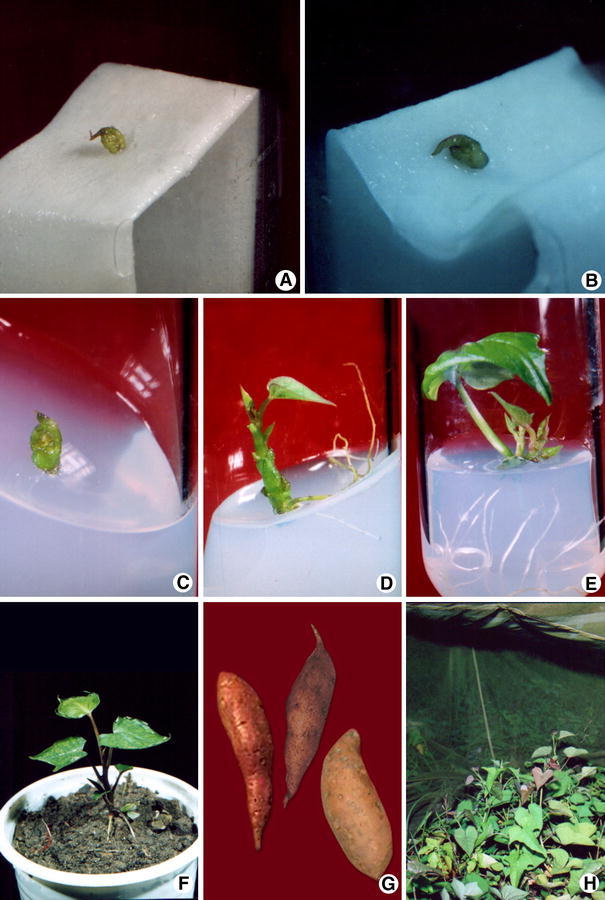
Viral diseases elimination through meristem culture in sweetpotato. **a** Development of isolated apical meristem (6 days old) on filter paper bridge in liquid MS medium. **b** Primary shoots initiation after 14 days in liquid medium. **c** Shoot with primary leaf development after subculturing in the semisolid medium. **d** Development of complete plantlet with root after transferring in a semisolid medium. **e** Multiplication of plantlets using excised nodal segment. **f** Acclimatization of plantlets. **g** Normal storage root developed in meristem-derived plants. **h** Meristem-derived plantlets in net house for producing clone from pre-original seed

Vine cuttings of the seven sweetpotato cultivars, viz, BARI-1, BARI-2, BARI-3, BARI-4, BARI-5, BARI-6 and BARI-7, collected from Tuber Crop Research Center, Bangladesh Agricultural Research Institute, Joydebpur and Regional Station, Bogra, were maintained in the Botanic Garden, Rajshahi University, from which explants were collected. Excised shoot tips collected from actively growing twigs were washed under running tap water and disinfected with 0.1 % mercuric chloride solution containing approximately 0.02 % Tween-20 [polyoxyethelene (20) sorbitan, oleate] for 6 min inside a running laminar air flow cabinet. Treated explants were washed four to five times with sterile distilled water to remove the effect of the sterilizing agent. Shoot apical meristem consisting of the apical dome with one to two leaf primorida was isolated using sterile hypodermic needle and scalpel under a dissecting microscope (Olympus) as described previously (Alam et al. 2004, 2010). To avoid dehydration, isolated meristems (0.3–0.5 mm) were transferred quickly on the filter paper bridge in test tubes containing sterilized liquid MS medium (Murashige and Skoog 1962) supplemented with GA_3_ and Kin either singly or in combination (Table 1). Carbon sources and concentrations were also optimized for primary establishment of the isolated meristems (Table 2). After 4 weeks, the developed meristems were subcultured on semisolid medium with different levels of plant growth regulator for the following 4–6 weeks for shoot elongation and root formation (Fig. 1c). Each mericlone was labeled as different lines. The developed mericlones were further multiplied using nodal segments (Fig. 1e). Some of the plantlets from each line were established up to at least five nodes development in soil for virus indexing. After indexing, only virus-negative mericlones (corresponding lines maintained in vitro) were subjected to massive multiplication for field trial.

**Table 1:** Effect of different concentrations and combinations of Kin and GA_3_ in MS medium for primary establishment of apical meristem of shoot tips from field-grown plants

PGR(mg/l)	Parameters	Cultivars						
		BARI-1	BARI-2	BARI-3	BARI-4	BARI-5	BARI-6	BARI-7
*Kin*								
1.0	Survival (%)	50.0	41.7	41.7	50.0	45.8	37.5	41.7
	Average vigor^a^	0.73	0.63	0.64	0.67	0.61	0.55	0.58
2.0	Survival (%)	66.7	54.2	54.2	62.5	54.2	45.8	50.0
	Average vigor	0.79	0.73	0.75	0.76	0.72	0.67	0.70
2.5	Survival (%)	70.8	62.5	62.5	66.7	62.5	54.7	58.3
	Average vigor	0.81	0.77	0.72	0.80	0.69	0.65	0.68
3.0	Survival (%)	58.3	50.0	50.0	54.2	50.0	41.7	45.8
	Average vigor	0.76	0.72	0.70	0.77	0.66	0.64	0.65
*GA*_*3*_								
1.0	Survival (%)	45.8	33.3	37.5	41.7	37.5	33.3	33.3
	Average vigor	0.63	0.61	0.58	0.63	0.56	0.51	0.53
1.5	Survival (%)	54.2	54.2	45.8	50.0	45.8	41.7	41.7
	Average vigor	0.72	0.69	0.63	0.80	0.62	0.58	0.60
2.0	Survival (%)	62.5	50.0	54.2	58.3	54.2	45.8	45.8
	Average vigor	0.77	0.71	0.68	0.73	0.66	0.60	0.62
3.0	Survival (%)	50.0	41.7	41.7	45.8	41.7	37.5	37.5
	Average vigor	0.67	0.65	0.60	0.67	0.59	0.56	0.58
*Kin* *+* *GA*_*3*_								
2.0 + 0.1	Survival (%)	66.7	66.7	62.5	62.5	58.3	54.2	58.3
	Average vigor	0.81	0.75	0.73	0.77	0.70	0.66	0.69
2.0 + 0.5	Survival (%)	79.2	75.0	70.8	70.8	66.7	62.5	62.5
	Average vigor	0.87	0.81	0.80	0.84	0.77	0.71	0.75
2.5 + 0.1	Survival (%)	62.5	58.3	58.3	54.2	58.3	50.0	50.0
	Average vigor	0.78	0.73	0.71	0.75	0.69	0.65	0.66
2.5 + 0.5	Survival (%)	75.0	62.5	66.7	66.7	62.5	58.3	54.2
	Average vigor	0.84	0.80	0.76	0.82	0.75	0.72	0.70

Data were recorded after 4 weeks of inoculation

^a^Calculation described in “Materials and methods”. Survival rate and average vigor were calculated from three independent experiments, each consisting of at least eight meristems

**Table 2:** Effect of carbon sources and their concentrations on primary establishment of apical meristem

Sources and concentrations	Average vigor^a^						
	BARI-1	BARI-2	BARI-3	BARI-4	BARI-5	BARI-6	BARI-7
*Sucrose* (%)							
3	0.87	0.81	0.80	0.84	0.77	0.72	0.75
4	0.94	0.90	0.88	0.93	0.87	0.81	0.83
5	0.85	0.75	0.71	0.78	0.70	0.70	0.70
6	0.66	0.60	0.61	0.63	0.59	0.53	0.55
*Table sugar* (%)							
3	0.76	0.72	0.70	0.75	0.65	0.62	0.63
4	0.87	0.82	0.81	0.86	0.70	0.65	0.67
5	0.81	0.72	0.67	0.75	0.67	0.60	0.64
6	0.65	0.55	0.57	0.60	0.53	0.61	0.60
*Maltose* (%)							
3	0.64	0.62	0.59	0.65	0.59	0.46	0.50
4	0.76	0.71	0.70	0.75	0.66	0.61	0.63
5	0.60	0.56	0.54	0.58	0.51	0.45	0.46
6	0.48	0.40	0.39	0.45	0.37	0.36	0.38
*Glucose* (%)							
3	0.54	0.56	0.52	0.56	0.52	0.50	0.50
4	0.62	0.60	0.58	0.63	0.56	0.53	0.55
5	0.47	0.51	0.50	0.51	0.50	0.42	0.46
6	0.42	0.34	0.34	0.42	0.35	0.30	0.34

Data were recorded after 4 weeks of inoculation

^a^Calculation described in “Materials and methods”. Average vigor was calculated from three independent experiments, each consisting of at least eight replications

To prepare tissue culture media, the pH was adjusted to 5.7, followed by autoclaving at 121 °C for 20 min (1.06 kg cm^−2^). For preparing semisolid medium, 0.8 % agar (w/v), (BHD, England) was added after adjusting the pH. Cultures were maintained at 25 °C under a 16-/8-h (light/dark) photoperiod with a light intensity of 50–60 μmol m^−2^ s^−1^ supplied by cool-white fluorescent lamps.

### Virus indexing of meristem-derived plantlets through grafting

Indicator plants (*I. setosa*) were grown from seed and maintained in a net house for using as root stock. Single node cutting of meristem-derived plants having a fully expanded leaf (scion) was wedge grafted on to 3-week-old indicator plant. Scalpels were sterilized with 70 % ethanol prior to cutting plant material to avoid cross-contamination of plants during the grafting process. The graft joints were wrapped with parafilm to prevent desiccation. The grafted plants were kept in growth chamber maintained at 25 ± 1 °C with a light intensity of 140–160 μmol m^−2^ s^−1^ for 5–7 days. After that, they were kept in open sunlight in a net house and observed for disease symptoms for 2 months. Four to six independently grown mericlones were graft tested for each cultivar. For comparison, some field-grown infected scions were also grafted.

### Acclimatization and field trials

After 3 weeks of acclimatization (Fig. 1f), micropropagated plants (*R*_1_) were transferred to experimental plots for evaluation in two conditions, namely net house and open field up to maturity. No insecticides were used for controlling viral vectors in open field condition. All necessary fertilizer applications and other cultural practices were followed. The storage root collected (Fig. 1g) from the respective net house and open field *R*_1_ plants were planted in the next season for producing enough vines for the *R*_2_ trial (Fig. 1h). The *R*_2_ generation was tested using planting materials from both open field and net house conditions using a split plot design with the field condition of source plant material (net house, open field and control) as main plot factor and the cultivars as subplot factor. Each treatment consisted of a total of 12 plants. Unlike *R*_1_, in the *R*_2_ trial traditional planting materials (not tested for viral diseases) were used as control.

### Data recording

Percentage of meristems showing growth response, average degree of meristem vigor, number of shoots per explant and number of roots per shoot were recorded during meristem culture establishment and micropropagation stage. The average degree of meristem vigor was calculated from visual observation, using a hypothetical 0.00–1.00 scale where 0.00 = no growth, 0.25 = poor growth, 0.5 = moderate growth, 0.75 = good growth and 1.0 = excellent growth. Each in vitro treatment consisted of at least eight replications and the entire in vitro experiment was repeated thrice. In the field trial, meristem-derived plantlets of *R*_1_ and *R*_2_ generation were planted in a split plot design with four replications. The length of main vine in centimeters, number of storage roots per plant and the root yield per plant (gm) from three randomly selected plants were evaluated to test their performance. Analysis of variances was performed for these yield-related characters using SAS Statistical Package version 9.1 (SAS Institute, Cary, NC, USA). Finally, the frequency of viral disease symptoms (%) was noted based on visual observation. Twelve plants of each cultivar were chosen for checking viral disease symptoms.

## Results

### Primary establishment of isolated meristem

#### Effect of growth regulators

The results on meristem culture in surface-sterilized shoot tip in liquid medium are presented in Table 1. Initial growth of the cultured meristem started within 6–15 days, as indicated by increasing size (vigor) and changing color to light greenish or pink depending on the cultivar (Fig. 1a). Growth and shoot (and sometimes root) development continued, resulting in the primary establishment of meristem (Fig. 1b). For this, MS medium supplemented with 2.0 mg l^−1^ Kin plus 0.5 mg l^−1^ GA_3_ showed the most vigorous response for all the studied cultivars. In this combination, about 79 % of excised meristems responded with an average vigor of 0.87 in cultivar BARI-1. Considerable growth response was also observed in MS medium containing 2.5 mg l^−1^ Kin irrespective of cultivar. Meristems failed to develop further when cultured in growth regulator-free medium. A varied degree of unexpected callus formation was observed when BAP was used in the medium and considered unsuitable for these cultivars (data not shown). Regarding meristem size, those smaller than 0.3 mm did not survive and 0.3–0.5 mm-sized meristems were used.

#### Optimization of carbon sources and their concentration

With the objective of enhancing the growth of the cultured meristem, four different carbon sources in four different concentrations were tested in MS medium containing 2.0 mg l^−1^ Kin plus 0.5 mg l^−1^ GA_3_ and the results are presented in Table 2. Among them, sucrose at 4 % level was found to be most effective. Increased sugar concentration shows poor penetration. After sucrose, commercial table sugar was also found to be better, followed by maltose and glucose. However, such distinct effect of sucrose concentration was not observed during the later phase of development for shoot and root formation and even in micropropagation of plantlets (data not shown). The effects of saccharides were generally similar across the cultivars.

### Shoot and root development from the primarily established meristem on semisolid medium

Overall, about 65–72 % of the meristem-derived tiny shoots showed further development when transferred to semisolid medium containing 2.5 mg l^−1^ Kin plus 0.5 mg l^−1^ GA_3_ (Fig. 1d), GA_3_ (2.0 mg l^−1^) or Kin (2.5 mg l^−1^) (data not shown). Spontaneous rooting was observed in all the cases. Those plantlets were multiplied by node cutting and were ready for virus assay before the massive micropropagation program.

### Clonal multiplication of plantlets

Following virus indexing, the remaining in vitro plant lines were used for massive micropropagation of plantlets and the results are presented in Table 3. The maximum number of shoots per explants was found in 3.0 mg l^−1^ Kin plus 0.5 mg l^−1^ GA_3_ containing medium irrespective of cultivars, while the maximum number of roots was obtained in the medium containing 3.0 mg l^−1^ Kin plus 1.0 mg/l^−1^ NAA.

**Table 3:** Effect of different combinations of Kin and GA_3_ in MS medium for multiplication of graft-tested mericlones using nodal explants

PGR (mg/l)	Parameters	Cultivars						
		BARI-1	BARI-2	BARI-3	BARI-4	BARI-5	BARI-6	BARI-7
*Kin*								
2.5	No. of shoot	4.29 ± 0.21	3.95 ± 0.25	3.75 ± 0.26	4.16 ± 0.27	3.62 ± 0.26	3.12 ± 0.20	3.66 ± 0.19
	Shoot length	5.21 ± 0.29	4.95 ± 0.29	4.54 ± 0.16	5.17 ± 0.17	4.30 ± 0.21	4.96 ± 0.16	5.28 ± 0.19
	No. of root	15.83 ± 0.39	15.00 ± 0.40	13.16 ± 0.46	12.50 ± 0.52	14.58 ± 0.55	13.87 ± 0.50	14.00 ± 0.50
3.0	No. of shoot	4.62 ± 0.25	4.50 ± 0.30	4.37 ± 0.29	4.62 ± 0.27	4.16 ± 0.22	3.91 ± 0.22	4.08 ± 0.24
	Shoot length	5.97 ± 0.22	5.41 ± 0.24	5.19 ± 0.14	5.48 ± 0.14	5.10 ± 0.23	4.92 ± 0.17	5.23 ± 0.23
	No. of root	18.12 ± 0.52	17.70 ± 0.43	15.16 ± 0.48	14.70 ± 0.57	17.95 ± 0.56	16.41 ± 0.61	14.54 ± 0.41
4.0	No. of shoot	3.41 ± 0.24	3.40 ± 0.27	3.33 ± 0.27	3.50 ± 0.26	4.37 ± 0.19	2.87 ± 0.21	3.16 ± 0.20
	Shoot length	5.42 ± 0.13	5.04 ± 0.26	5.19 ± 0.11	5.32 ± 0.19	4.63 ± 0.25	5.18 ± 0.18	4.55 ± 0.17
	No. of root	16.41 ± 0.52	13.54 ± 0.49	12.91 ± 0.46	16.66 ± 0.63	13.08 ± 0.39	15.08 ± 0.77	14.62 ± 0.36
*Kin* *+* *GA*_*3*_								
2.5 + 0.5	No. of shoot	4.83 ± 0.25	4.78 ± 0.53	4.66 ± 0.22	5.08 ± 0.32	4.50 ± 0.32	4.37 ± 0.24	4.29 ± 0.24
	Shoot length	7.40 ± 0.17	6.25 ± 0.16	5.55 ± 0.15	7.00 ± 0.28	5.36 ± 0.27	5.30 ± 0.13	5.32 ± 0.20
	No. of root	14.37 ± 0.53	12.87 ± 0.43	12.62 ± 0.63	14.37 ± 0.59	12.04 ± 0.42	13.62 ± 0.53	12.45 ± 0.42
3.0 + 0.5	No. of shoot	5.66 ± 0.28	5.60 ± 0.29	5.37 ± 0.30	5.83 ± 0.35	5.20 ± 0.28	5.12 ± 0.26	5.20 ± 0.27
	Shoot length	7.74 ± 0.18	7.24 ± 0.19	6.50 ± 0.15	7.74 ± 0.20	6.91 ± 0.32	6.40 ± 0.15	6.39 ± 0.18
	No. of root	17.50 ± 0.42	15.45 ± 0.45	13.79 ± 0.66	15.83 ± 0.61	16.33 ± 0.54	13.08 ± 0.70	15.70 ± 0.34
3.0 + 1.0	No. of shoot	3.41 ± 0.20	3.36 ± 0.50	3.16 ± 0.28	3.54 ± 0.26	3.08 ± 0.19	2.79 ± 0.19	2.66 ± 0.23
	Shoot length	6.77 ± 0.16	5.16 ± 0.15	5.80 ± 0.23	6.20 ± 0.22	5.55 ± 0.26	5.42 ± 0.15	5.22 ± 0.18
	No. of root	11.95 ± 0.44	12.20 ± 0.40	10.54 ± 0.41	14.75 ± 0.54	14.37 ± .52	12.04 ± 0.64	13.79 ± 0.44
*Kin* *+* *NAA*								
2.5 + 0.5	No. of shoot	2.66 ± 0.00	2.51 ± 0.53	2.45 ± 0.20	3.16 ± 0.81	2.16 ± 0.20	2.16 ± 0.17	2.12 ± 0.19
	Shoot length	4.79 ± 0.17	4.57 ± 0.21	4.17 ± 0.13	4.57 ± 0.18	3.67 ± 0.16	3.65 ± 0.12	3.84 ± 0.18
	No. of root	23.25 ± 0.90	20.62 ± 0.64	19.58 ± 0.86	18.41 ± 0.83	19.70 ± 0.64	17.58 ± 0.45	19.75 ± 0.51
3.0 + 0.5	No. of shoot	2.41 ± 0.17	2.45 ± 0.29	2.79 ± 0.23	3.25 ± 0.22	2.00 ± 0.19	2.08 ± 0.14	3.51 ± 0.18
	Shoot length	5.35 ± 0.15	4.72 ± 0.19	4.67 ± 0.17	4.90 ± 0.18	4.33 ± 0.23	4.31 ± 0.16	3.90 ± 0.26
	No. of root	20.20 ± 0.84	17.20 ± 0.61	17.16 ± 0.40	17.83 ± 0.70	18.91 ± 0.53	15.87 ± 0.78	17.20 ± 0.45
3.0 + 1.0	No. of shoot	2.04 ± 0.21	2.10 ± 0.50	1.91 ± 0.17	2.33 ± 0.16	1.58 ± 0.14	1.50 ± 0.15	1.62 ± 0.15
	Shoot length	4.58 ± 0.14	4.19 ± 0.16	3.86 ± 0.16	4.35 ± 0.16	3.57 ± 0.17	3.47 ± 0.13	3.43 ± 0.14
	No. of root	25.25 ± 0.80	25.33 ± 0.88	20.16 ± 0.60	19.08 ± 0.63	22.12 ± 0.61	18.91 ± 0.70	20.66 ± 0.33

Data were recorded after 4 weeks of inoculation

Shoot lengths were measured in centimeters. The data represent the mean values and SE of three independent experiments, each consisting of at least eight replications

### Virus indexing by grafting method

**Fig. 2:**
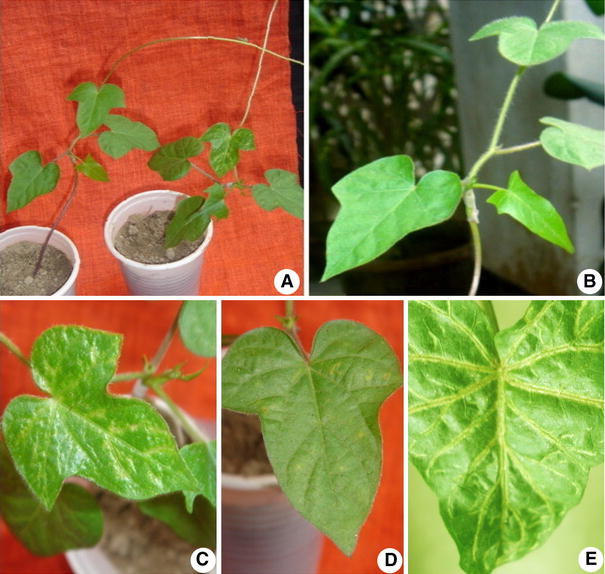
Representative picture of virus indexing of mericlones through the grafting method. **a** Field-grown and mericlone scion grafted on *I. setosa* stock. **b** Mericlones grafted on *I. setosa* stock showing no disease symptoms. **c**–**e** Virus-associated symptoms observed on *I. setosa* leaves upon grafting of field-grown sweetpotato plant including chlorosis, necrotic spots, vein chlorosis and slight cupping with rugosity

Around 85 % of the mericlones grafted showed no disease symptoms on *I. setosa* (Fig 2b). In contrast, most non-tested field samples (including those which were used as explant source) induced virus symptoms after grafting as assumed. The distinct symptoms on *I. setosa* include: small chlorotic spots, large veinal chlorosis, small veinal chlorosis, crinkling, leaf clearing and slight cupping with rugosity (Fig. 2a, c–e). Symptoms similar to single infections of SPFMV or SPMMV on *I. setosa* were most prominent after 3–5 weeks of graft inoculation. However, symptom like leaf rolling virus infections was not easily distinguished in *I. setosa*. On the other hand, multiple infections were correlated with severe and persistent symptoms (Fig. 2e). Nevertheless, to confirm the presence of specific virus(es), molecular diagnosis is required.

### Field trial of *R*_1_ and *R*_2_ plants

The results of field evaluation are presented in Tables 4 and 5. On comparing *R*_1_ managed either in net house or in open field, no marked variation was observed for yield-related characters. The analysis of variances further reveals that root yield of the *R*_2_ generation of net house-managed planting material was slightly better than both the open field and control plants. Other morphological characters were also more vigorous than both the open field and control plants. No varietal differences were observed for changing the field conditions. However, to test the actual yield effects, larger scale field trial is necessary. Viral disease incidence in the *R*_1_ net house plants was very low, whereas for the same in open field condition it was around 7 %. Similar trends were observed when tubers of both conditions were planted for vine production for the *R*_2_ trial (data not shown). The incidence was found to be higher for both the conditions in the *R*_2_ generations than *R*_1_. SPFMV reinfection rate was 3–8 and 6–13 % in net house and open field conditions, respectively. Among the symptoms (SPFMV, SPMMV and complex infections), the presence of SPFMV-like symptoms was found to be the highest.

**Table 4:** Field evaluation of *R*_1_ and *R*_2_ plants for yield components and disease incidence

Parameter	Cultivar														
	Plant material source	BARI-1		BARI-2		BARI-3		BARI-4		BARI-5		BARI-6		BARI-7	
		*R*_1_	*R*_2_	*R*_1_	*R*_2_	*R*_1_	*R*_2_	*R*_1_	*R*_2_	*R*_1_	*R*_2_	*R*_1_	*R*_2_	*R*_1_	*R*_2_
Length of main vine (cm)	Net house	73.3 ± 0.89	117.8 ± 0.67	78.3 ± 1.2	112.06 ± 0.76	70.5 ± 1.04	82.2 ± 1.48	68.2 ± 0.44	190.9 ± 0.62	70.3 ± 1.33	202.9 ± 1.12	74.0 ± 0.58	201.1 ± 0.78	66.7 ± 1.2	231.4 ± 0.85
	Open field	70.7 ± 1.67	116.7 ± 1.07	76.0 ± 1.00	111.8 ± 0.49	64.7 ± 1.76	81.1 ± 0.82	63.3 ± 1.20	189.2 ± 0.68	66.7 ± 1.20	202.2 ± 1.13	72.8 ± 1.17	200.0 ± 0.82	61.7 ± 0.88	230.4 ± 0.82
	Control	–	115.1 ± 1.25	–	111.2 ± 0.43	–	80.4 ± 1.16	–	189.6 ± 0.37	–	202.1 ± 0.34	–	200.3 ± 0.50	–	230.4 ± 1.14
Storage root no./plant ( ± SE)	Net house	1.67 ± 0.33	2.00 ± 0.47	2.33 ± 0.67	3.67 ± 0.27	3.67 ± 0.67	4.00 ± 0.00	4.66 ± 0.88	5.33 ± 0.27	4.67 ± 0.33	5.0 ± 0.00	3.00 ± 0.57	3.0 ± 0.47	2.67 ± 0.88	2.33 ± 0.54
	Open field	1.67 ± 0.67	2.00 ± 0.47	2.0 ± 0.57	3.33 ± 0.27	3.67 ± 0.88	3.6 ± 0.27	4.33 ± 1.2	5.33 ± 0.72	4.33 ± 0.67	5.33 ± 0.27	3.33 ± 0.88	2.67 ± 0.72	2.33 ± 0.33	2.33 ± 0.27
	Control	–	2.00 ± 0.81	–	3.67 ± 0.54	–	3.66 ± 0.72	–	5.00 ± 0.47	–	5.00 ± 0.47	–	2.66 ± 0.54	–	2.67 ± 0.27
Root yield/plant (g)	Net house	335 ± 2.6	538 ± 1.53	306 ± 1.5	576 ± 1.45	245 ± 1.45	374 ± 1.45	355 ± 1.5	482 ± 2.08	310 ± 1.00	520 ± 1.76	321 ± 1.15	567 ± 1.16	200 ± 2.02	338 ± 1.5
	Open field	334 ± 1.85	531 ± 0.88	310 ± 1.15	570 ± 0.88	238 ± 0.88	369 ± 1.53	352 ± 2.33	481 ± 0.67	312 ± 1.20	517 ± 1.20	322 ± 1.76	563 ± 2.40	210 ± 2.03	330 ± 1.15
	Control	–	529 ± 0.88	–	563 ± 1.85	–	363 ± 1.85	–	476 ± 0.88	–	511 ± 2.02	–	559 ± 0.57	–	325 ± 1.85
SPFMV-like symptoms (%)	Net house	0.0	5.9	0.0	6.1	0.0	3.0	0.0	0.0	0.0	3.5	0.0	5.6	0.0	0.0
	Open field	6.7	11.1	0.0	8.3	7.1	8.3	7.1	13.8	0.0	8.8	7.7	6.0	0.0	0.0
	Control	–	41.7	–	27.8	–	25.0	–	19.4	–	22.2	–	25.0	–	19.4
SPMMV-like symptoms (%)	Net house	0.0	0.0	0.0	3.0	0.0	3.0	0.0	2.7	6.7	3.6	0.0	2.7	6.6	8.3
	Open field	0.0	0.0	6.7	0.0	0.0	0.0	0.0	0.0	0.0	2.9	0.0	0.0	0.0	5.6
	Control	–	2.7	–	5.6	–	13.8	–	0.0	–	8.3	–	0.0	–	0.0
Complex infection-like symptoms (%)															
	Net house	0.0	0.0	0.0	0.0	0.0	0.0	0.0	0.0	0.0	0.0	0.0	2.7	0.0	0.0
	Open field	0.0	0.0	0.0	2.7	0.0	6.0	0.0	0.0	0.0	0.0	0.0	3.0	0.0	0.0
	Control	–	8.3		5.6	–	0.0	–	13.8	–	11.1	–	19.4	–	11.1

No control was used in the *R*_1_. *R*_2_ trial was conducted in open field condition; vines were obtained either from net house or from open field and compared with traditional cuttings

**Table 5:** Mean squares (MS) from the analysis of variance of yield and its components of seven varieties under three different field conditions in the R2 generation

Source of variation	*df*	Length of main vine (cm)	Root number/plant	Root yield/plant (g)
Replication	2	4.12	1.16	2.68
Field (A)	2	9.73	0.21	486.02**
Main plot error (Ea)	4	1.13	1.54	19.56
Variety (B)	6	29,629.31***	6.33**	80,389.51***
A × B	13	0.68	0.96	3.89
Sub plot error (Eb)	36	2.02	0.11	8.55

The asterisk ** or *** signifies *p* < 0.01 or *p* < 0.001, respectively

## Discussion

Plant development from isolated meristem usually requires exogenous hormonal supplement in culture medium. In our study, simultaneous use of Kin and GA_3_ was found to be good, as supported by earlier work (Love et al. 1989), while use of BAP or even auxins like NAA, IAA, and 2,4-D for the same was also reported. Sweetpotato has a very high genetic variability in its germplasm. Therefore, the differences in tissue culture response across the cultivars might be due to genotypic effect. Being tiny and free of conducting tissues, liquid culture medium is beneficial for growth and development of isolated meristem as found in our experiment and of other researchers (Elliott 1969; Alam et al. 2004). The advantage of liquid medium lies in easier availability of water and dissolved nutrients to the entire surface of the explants. However, the considerable number of isolated meristems which died might be due to injury during their isolation. The normal rate for death in this regard was reported as 25–40 % (Love et al. 1989). In the latter phase, during shoot and root development from primarily established meristems, combined use of Kin and GA_3_ was also found to be beneficial with a slightly higher concentration.

Plant cells and tissues in the culture medium lack autotrophic ability. Even tissues which are initially green or acquire green pigments under special conditions during the culture period are not autotrophs for carbon. Because of this, in most of the cases, the normal functions of the chloroplasts are either absent or blocked (Maretzki et al. 1974). Therefore, it is imperative to supply external carbon sources to produce enough carbohydrate in order to promote cell growth and subsequent regeneration. Meristem culture of sweetpotato was not exceptional; nevertheless, it was influenced by carbon sources. In general, sucrose is the carbohydrate of choice as carbon source for in vitro culture, probably because it is the major transport sugar of higher plants (Thompson and Thorpe 1987). However, a number of species can grow on carbohydrates different from sucrose (Marchal et al. 1992; Vu et al. 1993). Our results indicate that sucrose not only act as carbon source, but also as an osmoticum. The detrimental effect of using high concentration of sucrose (above 5 %) for meristem culture supported its role as an osmoticum. However, the effect was not observed during the later stages of development (micropropagation) probably due to osmotic adjustment of the cultured cells. Osmotic role of soluble sugars in cultured cells has already been reported (Lipavská and Vreugdenhil 1996).

Indicator plant *I. setosa*, susceptible to most known viruses of sweetpotato, has been used in virus-indexing systems in order to verify the presence of known viruses (Gutiérrez et al. 2003) and could be used in testing meristem-derived plants. The presence of SPFMV in *I. setosa* has been characterized by symptoms of vein clearing, chlorotic mottle, vein banding or small crinkled leaves, while leaf mottling, vein chlorosis, dwarfing and poor growth are common symptoms of SPMMV (Love et al. 1989). Our virus indexing in traditional non-tested material, in addition, indicates some other symptoms like cupping with rugosity, stunting, necrotic spots and bright veinal chlorosis, which might be due to interaction between SPFMV and others. Mixed infections of SPFMV with other potyviruses have also been reported by (Moyer and Salazar 1989). The apparent synergistic effect of SPFMV and SPCSV is now well documented (Gutierrez et al. 1999). Nevertheless, viral synergism is not exclusively restricted to SPFMV and SPCSV, as other virus interactions have been reported (Di Feo et al. 2000; Salazar and Fuentes 2000; Clark and Hoy 2006). The symptomatology seems to be different depending on the virus complex and is difficult to be distinguished by inexperienced observers.

The field performance of meristem-derived plants in both *R*_1_ and *R*_2_ generations did not change much under net house condition. The incidence of viral diseases in the traditional non-tested material was high as anticipated. The use of clean planting materials consistently produced higher storage yield than the farmers’ planting materials. Virtually, there was no trace of mixed infection (complex infection) from the net house plant. According to our results, reinfection under net house conditions is only a small possibility. Reinfection was observed under open field in both years, indicating that natural sources of infection occur. Reinfection of sweetpotato viruses depends upon various factors including vector availability, local cultural practices, disease incidence, etc. Up to 50 % reinfection and 30 % reduction in yield compared to virus-free control plants was reported by Milgram et al. (1996). The effectiveness of using net house has also been suggested to protect tomato mericlones from insect vectors under tropical condition in our previous work (Alam et al. 2004).

**Fig. 3:**
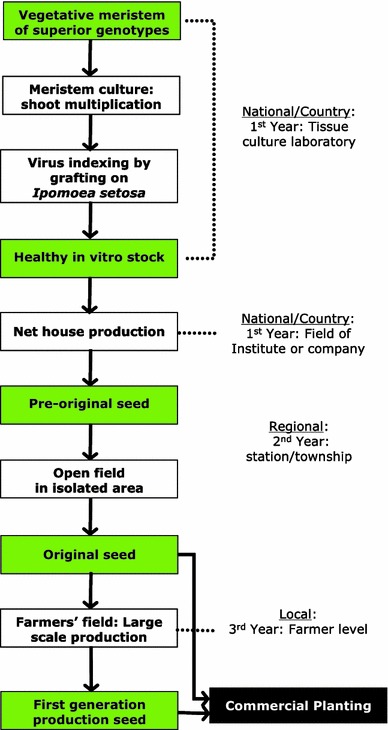
Flowchart showing a scheme for production of virus-free planting material for tropical environment from tissue culture laboratory to the farmer level

The results clearly showed that the medium, which is used for in vitro culture of other sweetpotato cultivars from different agro-ecological areas, is not exactly suitable for our cultivars due to the very high level of genetic diversity. After 1 month of culture, the plantlets had three to four nodes, which can be multiplied using single node cuttings. Thus, three to four new plantlets could be produced from a single nodal explant 1 month later. Assuming a monthly multiplication cycle from a four-node plantlet including around 10 % mortality, the potential number of planting materials would be about 3.8 million. This large number of pathogen-free material can provide significant economic benefits. From a practical viewpoint, it is therefore advisable to keep the field free of insect vectors in order to minimize reinfection every year. Based on our study, the entire protocol for commercial production of diseases-free cutting is presented in Fig. 3 in a flowchart. An extensive trial is necessary to fully assess the yield and economic benefit at the farmer level of using this seed system.
